# Metal–Curcumin Complexes in Therapeutics: An Approach to Enhance Pharmacological Effects of Curcumin

**DOI:** 10.3390/ijms22137094

**Published:** 2021-06-30

**Authors:** Sahdeo Prasad, Dan DuBourdieu, Ajay Srivastava, Prafulla Kumar, Rajiv Lall

**Affiliations:** 1 Research and Development Laboratory, Noble Pharma LLC, Menomonie, WI 54751, USA; 2 Research and Development Laboratory, Vets-Plus Inc., Menomonie, WI 54751, USA; dand@vets-plus.com (D.D.); drajay@vets-plus.com (A.S.); prafullak@vets-plus.com (P.K.); lallr@vets-plus.com (R.L.)

**Keywords:** metals, curcumin, inflammation, oxidative stress, chronic diseases

## Abstract

Curcumin, an active component of the rhizome turmeric, has gained much attention as a plant-based compound with pleiotropic pharmacological properties. It possesses anti-inflammatory, antioxidant, hypoglycemic, antimicrobial, neuroprotective, and immunomodulatory activities. However, the health-promoting utility of curcumin is constrained due to its hydrophobic nature, water insolubility, poor bioavailability, rapid metabolism, and systemic elimination. Therefore, an innovative stride was taken, and complexes of metals with curcumin have been synthesized. Curcumin usually reacts with metals through the β-diketone moiety to generate metal–curcumin complexes. It is well established that curcumin strongly chelates several metal ions, including boron, cobalt, copper, gallium, gadolinium, gold, lanthanum, manganese, nickel, iron, palladium, platinum, ruthenium, silver, vanadium, and zinc. In this review, the pharmacological, chemopreventive, and therapeutic activities of metal–curcumin complexes are discussed. Metal–curcumin complexes increase the solubility, cellular uptake, and bioavailability and improve the antioxidant, anti-inflammatory, antimicrobial, and antiviral effects of curcumin. Metal–curcumin complexes have also demonstrated efficacy against various chronic diseases, including cancer, arthritis, osteoporosis, and neurological disorders such as Alzheimer’s disease. These biological activities of metal–curcumin complexes were associated with the modulation of inflammatory mediators, transcription factors, protein kinases, antiapoptotic proteins, lipid peroxidation, and antioxidant enzymes. In addition, metal–curcumin complexes have shown usefulness in biological imaging and radioimaging. The future use of metal–curcumin complexes may represent a new approach in the prevention and treatment of chronic diseases.

## 1. Introduction

Curcumin, or diferuloylmethane, is a yellow crystalline hydrophobic polyphenol isolated from turmeric (*Curcuma longa*). It is one of the major components of the curcuminoid family, which also includes the two other curcuminoids, desmethoxycurcumin and bis-desmethoxycurcumin. Turmeric has been used as a spice in many southeast Asian countries as it provides distinctive color and flavor in food. However, curcumin is also used as a traditional medicine against various chronic diseases, including cardiovascular, neurodegenerative, respiratory, pulmonary, autoimmune, metabolic, and other various types of diseases. It is also found to be effective against anorexia, cough, coryza, hepatic diseases, and sinusitis [1,2]. The preventive and therapeutic effects of curcumin have been attributed to its pleiotropic pharmacological properties. Accumulated evidence suggests that curcumin has anti-inflammatory, antioxidant, wound healing, hypoglycemic, and antimicrobial properties [3]. Despite the tremendous biological activities of curcumin, it still has some physical inadequacies, including its water insolubility and poor bioavailability. In the last few decades, modifications to its structure, including synthesizing analogues of curcumin, have been performed to circumvent these problems.

**Figure 1:**
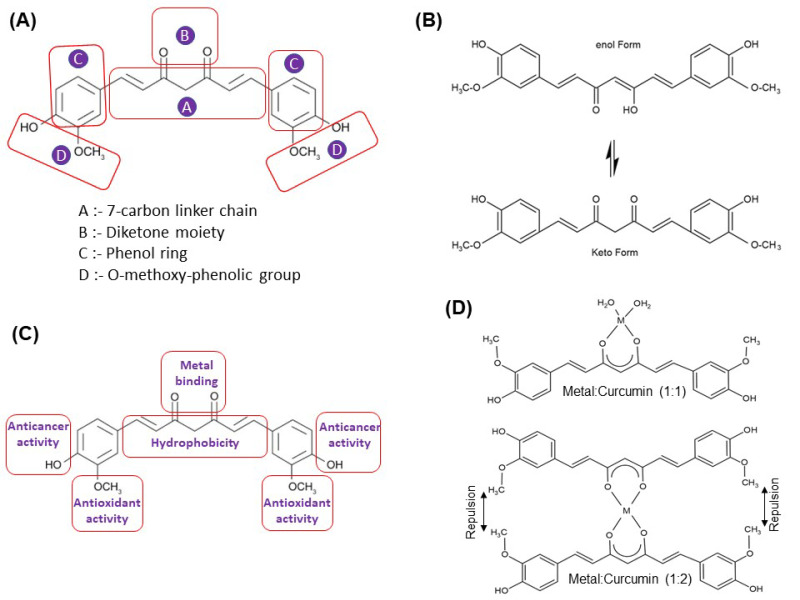
(**A**) The basic structure of curcumin. (**B**) Existence of curcumin in keto-enol tautomeric forms. (**C**) Groups of curcumin responsible for its biological properties. (**D**) Structure of 1:1 and 1:2 metal:curcumin complexes showing the β-diketone moiety of curcumin as the metal binding site.

Curcumin is a beta-diketone where feruloyl groups replace two hydrogens in the structure. It is known to exist in at least two tautomeric forms, keto and enol. The keto form of curcumin exists in acidic and neutral pH media, while the enol form exists in alkaline pH medium [4]. Structurally, curcumin is comprised of a seven-carbon linker and three major functional groups. These functional groups include an α, β-unsaturated β-diketone moiety, an aromatic O-methoxy-phenolic group, and a seven-carbon linker molecule (Figure 1A). Two α, β-unsaturated carbonyl groups connect the aromatic rings of curcumin. The diketones easily deprotonate themselves and form enolates (Figure 1B), whereas the α, β-unsaturated carbonyl acts as a Michael acceptor and undergoes nucleophilic addition [5]. The antioxidant activity of curcumin is caused by its phenolic group, whereas the carbon linker molecule provides hydrophobicity [6] (Figure 1C). As curcumin is hydrophobic in nature, curcumin has been structurally modified to increase its biological activity.

In recent decades, complexes of curcumin with various metals have been synthesized to overcome the issues associated with curcumin and to make it more biologically potent. Metals interact with the curcumin ligand, which brings a modification to curcumin’s overall structure and improves the biological efficacy of curcumin. It has been shown that the carbonyl group at the diketone moiety is destabilized due to the metal ion coordination [7]. Since metals are known as an enzyme coactivator, curcumin–metal complexes can interact with active sites of enzymes and induce multiple cellular processes. Although complexes of curcumin as nanoparticles, liposomes, micelles, and phospholipids have been developed that have shown improved biological efficacy [8], complexes of curcumin with transition metals may provide another approach to overcome the issues associated with curcumin.

## 2. Metal–Curcumin Interactions

**Figure 2:**
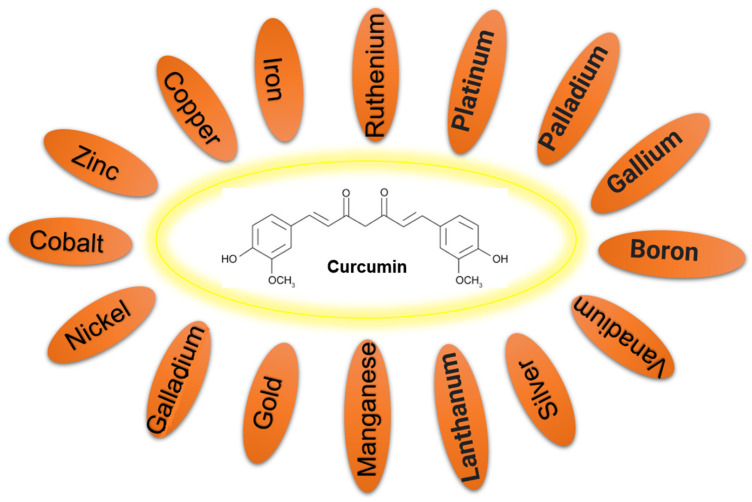
Curcumin interacts with various metals and forms metal–curcumin complex.

As the α,β-unsaturated β-diketo moiety of curcumin is reported to be a strong chelating agent, it interacts with various metal ions. Various techniques such as atomic force microscopy (AFM), UV-vis spectroscopy, Fourier transform infrared, nuclear magnetic resonance, and mass spectroscopy have been implemented to determine the binding of metals to the curcumin. Metals usually bind to the keto-enol (β-di-ketone moiety) group of curcumin by chelation. Chelation is a type of a chemical bonding process that involves the formation of dative covalent bonds (also known as coordinate bonds) between at least one multidentate ligand and a metal cation. While classic covalent bonds involve the sharing of electrons coming from both atoms, dative covalent bonds have the electrons provided by only one atom. The nature of this bond is of a semipolar. This chelation bond interaction induces a structural variation to the curcumin [9]. Normally, metals bind either one or two curcumin molecules; however, binding of three curcumin molecules has also been reported, e.g., an octahedral complex reported with Fe^3+^ [10]. The coordination of metals with curcumin occurs through its enolic group, as the enolic proton is replaced by a metal ion (Figure 1D), and the o-methoxy phenolic group remains intact in the complexes. The binding of one metal with curcumin in a 1:1 ratio causes a difference in all four metal-O bond lengths in the complex, while those are identical in the 1:2 metal–curcumin complex. Metal–curcumin (1:1) also causes orthorhombic symmetry in the structure, while square planar coordination has been observed around the metal in the 1:2 metal–curcumin complex. In addition, steric repulsion has been reported between the methoxy groups of the phenolic rings of both curcumins of the 1:2 metal–curcumin complex [11]. However, as shown in Figure 2, complexes with curcumin have been synthesized with many transition and nontransition metal ions, rare earth ions, and metal oxides.

There are about 80 elements that are considered metals in the periodic table. Some metals serve as a basis for common engineering purposes, some for medical purposes, and some for both. How they are used depends on their specific chemical and physical attributes, such as ionic state, physical geometry, valance bonding, and other chemical characteristics. These chemical characteristics differ from one metal to another but, at the same time, can be taken advantage of for various medical uses when creating curcumin–metal complexes. Creating a curcumin–metal complex not only affects the physical and chemical properties of curcumin, but it also affects the biological reactivity of the metals. In general, curcumin–metal complexes reduce the toxicity of metals [5]. The complexation with certain metals like Cu^2+^ and Mn^2+^ can make for new metal-based antioxidants [12,13].

## 3. Synthesis of the Curcumin–Metal Complexes

Curcumin acts as a ligand and forms stable complexes with almost all the metal ions and nonmetals. Thus far, different methodologies have been used to synthesize metal–curcumin complexes. In order to form a curcumin–metal complex, it is necessary to obtain the curcumin and the metal in a solution format in order for the reaction to occur. As curcumin is not water soluble to any great extent, the curcumin must be solubilized with various organic solvents. These solvents typically can include methanol, ethanol, and acetone. On the other hand, metals used in the reaction are typically in a salt format that can be solubilized in aqueous solutions. Some metal salts, such as ZnCl_2_, happen to be soluble in both water and certain organic solvents such as methanol. Depending on the metal, it is possible to have the chelation reaction in solvents that do not cause the problems for environmental issues that can occur with organic solvents. For example, Hieu et al. [14] synthesized metal–curcumin complexes by mechanical mixing of metal chloride salts and curcumin (metal ion:curcumin 1:1 mol with Zn^2+^; 1:3 with Fe^3+^) in a mortar until homogenous powder mixtures were obtained. A propylene glycol:water (1:1 *v*/*v*) solution was added to the mixtures followed by mechanical shaking and drying at 60 °C to obtain powdered complexes of curcumin metal ion. More traditional organic solvent approaches are seen with a curcumin complex with lanthanum metal being synthesized by dropping a curcumin solution in ethanol into a solution of La(NO_3_)_3_·6H_2_O (also in ethanol) at pH 6 followed by refluxing at 80 °C [15]. Similarly, a Pd–curcumin complex was synthesized by mixing a solution of curcumin and Na_2_CO_3_ in methanol, with a solution of PdCl_2_ (also in methanol) followed by heating of the mixture at 60 °C until a clear blood-red solution was formed [15]. The synthetic approach used will depend on the type of metal and the chemical characteristics of that particular metal. Various metal complexes with curcumin have been synthesized, but as we have seen, the method used will depend on the chemistry and physical characteristics of the individual metal.

## 4. Pharmacological Effects of Metal–Curcumin Complex

**Figure 3:**
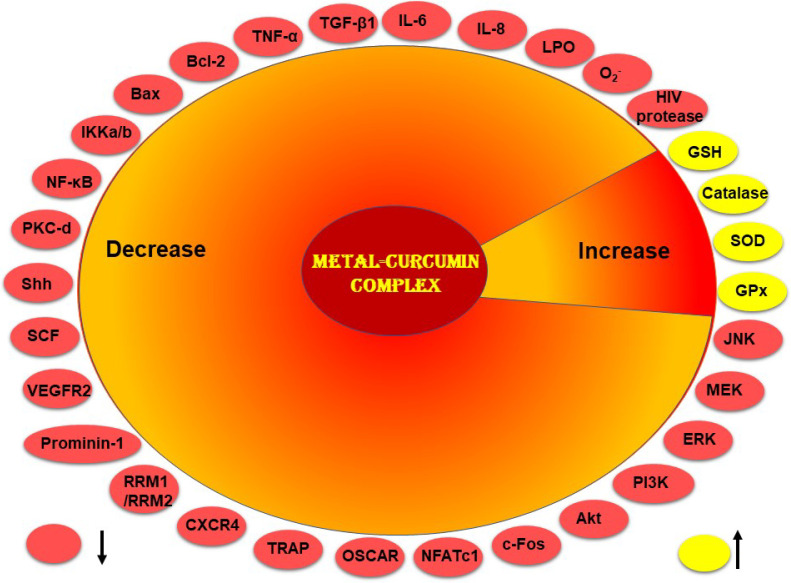
Curcumin–metal complex modulates biomarkers that are involved in health and diseases.

Curcumin–metal complexes show improved pharmacological effects compared to free curcumin [16]. These complexes have the ability to enhance antioxidant activity; inhibit inflammation; exert antimicrobial, antiviral, and anticancer effects; and exhibit neuroprotective and gastroprotective activities (Table 1). These activities have been mediated through the modulation of multiple molecular markers as shown in Figure 3.

### 4.1. Antioxidant

Metal–curcumin complexes are known to have multiple capabilities. As a result, they have been termed to have “multi-anti” actions [37], such as having various pharmacological activities including antioxidant actions that are better than either free curcumin or free metal ions alone. Curcuminoids, such as curcumin and its metabolite, tetrahydrocurcumin, have antioxidant capabilities that are useful for various health conditions [4]. Of the various herbs that possess antioxidant properties, curcumin is one of the most important ones, especially from a commercial standpoint. Curcumin is thought to be a phenolic chain-breaking antioxidant that donates H^+^ atoms from the phenolic group [38]. In addition, curcumin has the ability to enhance the activities and levels of antioxidant enzymes, including catalase, superoxide dismutase, and glutathione peroxidase, and to quench free radical generation [39]. This has led to numerous preclinical and clinical trials to show its efficacy. Indeed, curcumin supplementation in humans has been shown to have significant antioxidant capacity and a tendency to decrease malondialdehyde (MDA) concentrations [40].

As oxidative stress is caused by the imbalance of free radicals and antioxidant status of the body, metal–curcumin complexes manage oxidative stress either by reducing free radicals, enhancing antioxidant levels, or both. Most research indicates that curcumin–metal complexes have improved antioxidative ability over free curcumin alone [7,14,41,42]. Curcumin complexes with Cu^2+^, Zn^2+^, Mn^2+^, Mg^2+^, and Fe^2+^ have shown better DPPH radical scavenging and ferrous-reducing power activities than free curcumin at the same dosage. One study showed that the metal:curcumin ratio (1:1) is more active in scavenging superoxide anion radicals (through proton transfer or electron transfer) but less active in scavenging DPPH radicals (through H-atom transfer) than the metal:curcumin ratio (1:2) [11]. Zinc appears to possess an increased ability to enhance antioxidant activity when complexed to curcumin. This may be due to zinc’s ability to stabilize the interaction energies of metal complexes with free radicals better than manganese or iron [7]. In an animal model, it has been found that the administration of a Zn^2+^–curcumin complex reverses the decreased activities of the antioxidant enzymes and increases the MDA level caused by cold-restraint stress [17]. Another complex of Fe^2+^–curcumin not only retained antioxidant enzyme activity after the chelation but also improved the antioxidant activity of curcumin [18]. Thus, this complex has the potential to act as a therapeutic agent by reducing oxidation.

Curcumin-capped gold nanoparticles and curcumin–silver nanoparticles have also shown excellent antioxidant activity as shown by the DPPH radical test [19,20]. It has been shown that the curcumin–Cu^2+^ complex possesses increased superoxide scavenging activities when compared with free curcumin. Additionally, curcumin–Cu^2+^ or –Zn^2+^ complexes enhance the activities of antioxidant enzymes, such as catalase, superoxide dismutase, and glutathione peroxidase, and attenuate the rise of MDA levels in pheochromocytoma (PC12) rat neuronal cells [21]. The Cu^2+^–curcumin complex has also been demonstrated to exhibit strong scavenging activity of DPPH radicals [11]. Antioxidant activities of another curcumin complex synthesized with chitin-glucan-based zinc oxide nanoparticles were estimated using a DPPH free radical scavenging assay and ABTS+ assay. It was found that the loading of curcumin into zinc oxide nanoparticles resulted in increased antioxidant activity of the complex [22]. Moreover, using DPPH and ABTS+ assays, it was shown that complexes of ruthenium metal with curcumin have potent antioxidant activities as compared to doses of curcumin alone [23]. The antioxidant effect of Mn^2+^–curcumin complex has also been determined in cadmium-intoxicated mice. It has been observed that Mn–curcumin complexes and unchelated curcumin prevent the increase of hepatic lipid peroxidation and restore hepatic glutathione (GSH) levels, although no potentiation was observed [43]. Along with curcumin, the antioxidant activity of its derivative diacetylcurcumin–gallium complex has been studied, and it was shown that it offers an increase in antioxidant efficiency [24]. Further studies also confirmed the antioxidant activities of diacetylcurcumin–metal complexes. The diacetylcurcumin–metal (Mg, Zn, Cu, and Mn) complexes have exerted good antioxidant effects, as evident by decrease in lipid peroxidation [44].

**Figure 4:**
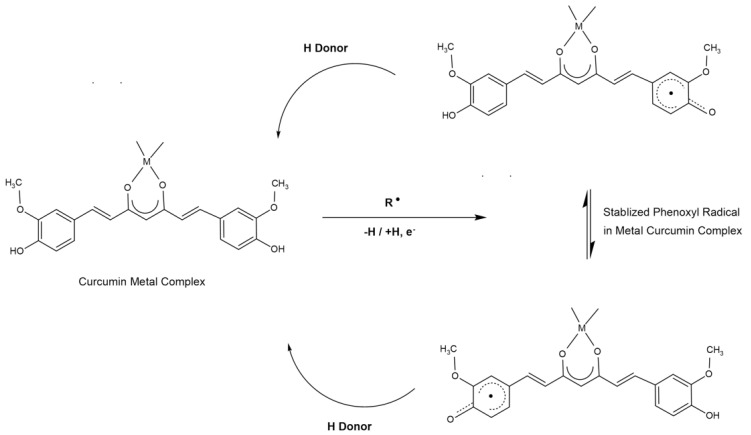
Free radical binding mechanism to the metal–curcumin complexes.

One reason why curcumin–metal complexes appear to be more potent antioxidants is apparently due to metal stabilizing curcumin in the complex [11] when a superoxide radical binds to it. When a metal, such as copper, complexes with curcumin, the complex can sustain the distortion from a square-planar geometry to a distorted tetrahedral geometry when it reacts with a superoxide radical. Curcumin has the property of becoming easily oxidized and subsequently changing its structure when free radicals bind. However, the binding of metal ions to the curcumin does not lead to much change in the physical geometry of the curcumin and thus allows a curcumin–metal complex to remain intact. The metal stabilizes the curcumin structure when free radicals bind to the phenolic structure of the curcumin. When a free radical encounters the phenolic part of curcumin, the electron is distributed around the phenolic ring structure but at the same time will distort the overall structure of the curcumin molecule. The metal seems to help stabilize this distortion of the overall structure, and this helps the curcumin–metal complex accepts the extra free radical electron even better than plain curcumin alone (Figure 4). This results in the occurrence of many redox cycles of curcumin and the exhibition of better antioxidant activity. The phenolic structure of a curcuminoid appears to be the site where free radicals interact [41] with minimal involvement of the keto-enol moiety. This is consistent with the idea that aromatic ring structures of antioxidants have the ability to delocalize unpaired electrons and thus help terminate the free radical aspects.

### 4.2. Anti-Inflammatory

Curcumin is well known as an anti-inflammatory agent, as it was observed to inhibit inflammatory mediators in preclinical and clinical models. Curcumin modulates various inflammatory cytokines, chemokines, inflammatory transcription factors, inflammatory enzymes, and proteases [2,45]. However, an enhanced anti-inflammatory activity of curcumin has been observed in metal–curcumin complexes. It has been observed that the binding of metals significantly alters the chemical properties of the curcumin, which leads to significant changes in the anti-inflammatory efficacy of the complexes.

Several metal–curcumin complexes have been synthesized, and their anti-inflammatory activity has been examined using in vitro and animal models. In one study, a complex of Zn^2+^–curcumin was synthesized and its biological activities, including anti-inflammatory activity, were determined. It was found that treatment with Zn^2+^–curcumin complex inhibits pylorus-ligature-induced inflammatory markers NF-kappaB (NF-κB), transforming growth factor beta(1) (TGF-β1), and interleukin (IL)-8 in rats [26]. Furthermore, oral treatment of Zn^2+^–curcumin inhibited the levels of proinflammatory cytokines, such as tumor necrosis factor-α (TNF-α) and IL-6, and H^+^-K^+^-ATPase was inhibited in the mucosa of rats exposed to ethanol [25]. Thus, these results indicated that the Zn^2+^–curcumin complex inhibits various inflammatory pathways.

Other complexes of metals with curcumin have also been used for reduction of inflammation. Curcumin–ferrous complexes have shown strong anti-inflammatory activity in experimental models. In a rat paw edema model, a curcumin–iron complex was found to reduce edema by 59.76%, whereas free curcumin was able to reduce edema by 51.32%. This effect of the reduction of edema is comparable to the standard drug indomethacin, which induced a 61.76% reduction [46]. A Cu^2+^–curcumin complex has been shown to have superior biological activity compared to curcumin alone. In terms of anti-inflammatory activity, a Cu^2+^–curcumin complex was found to be better in the modulation of the irradiation-induced activation of PKC delta and NF-κB suppression [27]. It has been also demonstrated that curcumin–Cu^2+^ or –Zn^2+^ complexes downregulate the hydrogen peroxide (H_2_O_2_)-induced NF-κB inflammatory pathway in PC12 neuronal cells, which further leads to the inhibition of cell apoptosis. Thus, these complexes possess significant neuroprotective effects [21]. The complex of curcumin with gadolinium (curcumin–PEGylated α-Gd2(MoO4)3) not only inhibited the proliferation of cancer cells, but also inhibited inflammatory markers. In human pancreatic cancer cell lines, this complex showed increased inhibition of inflammatory mediators pIKKα, pIKKα/β, and NF-κB as compared to pure curcumin [28]. These findings suggest that producing complexes of metals with curcumin would be useful as a nutraceutical in fighting inflammation.

### 4.3. Antimicrobial

It is well known that curcumin has antimicrobial activity against a wide range of bacterial species. The sensitivity of curcumin towards Gram-positive bacteria has been shown to be higher than Gram-negative bacteria [47]. Interestingly, the antimicrobial activity of curcumin can be further enhanced by exposure to light. However, to further increase the antimicrobial activity of curcumin, a complex with metal or curcumin nanoparticles was developed, which considerably increased its activity [48,49]. It has been observed that metal–curcumin-conjugated DNA complexes display a prolonged lag phase in bacterial growth curves of *Escherichia coli* and *Bacillus subtilis* [9], indicating their bacterial growth suppressing effects. Curcumin complexes with Co^2+^, Ni^2+^, and Cu^2+^ ions were also assessed for their antibacterial activity against *Staphylococcus aureus*, *Escherichia coli*, *Klebsiella pneumoniae*, *Pseudomonas aeruginosa*, and *Streptococcus pyogenes*. The Cu^2+^–curcumin complex showed the highest antibacterial activity. However, the relative order of antibacterial activity against *S. Pyogenes*, *S. aureus*, and *E. coli* was Cu^2+^ > Ni^2+^ > Co^2+^ > (L), while with *P. aeruginosa*, *K. pneumoniae*, the order of activity was Cu^2+^ > Co^2+^ > Ni^2+^ > (L). These complexes also exerted anthelmintic activity against *Pheretima posthuma*. Interestingly, the Cu^2+^–curcumin complex was most effective when followed by Co^2+^ and Ni^2+^ [29].

A Zn^2+^–curcumin complex was shown to exhibit dramatic toxicity against *P. aeruginosa* [30]. Similarly, a curcumin complex with chitin glucan-zinc nanomaterial showed excellent antibacterial activity [22]. Ru^2+^–curcumin complex has been reported to exhibit antimicrobial activity, particularly with the drug-resistant Gram-positive *S. aureus*. This complex exerted inhibitory activity and high selectivity against a wide variety of methicillin- and vancomycin-resistant *S. aureus* strains. Moreover, this complex decreased mean bacterial counts in a murine model of Staphylococcus infection compared to vancomycin. These observations indicate that the Ru^2+^–curcumin complex has a good antimicrobial potential in both in vitro and in vivo models [31].

The Mn^2+^–curcumin complex has been shown to possess significant antibacterial activity against both Gram-negative (*E. coli*) and Gram-positive (*S. aureus*) bacteria compared to free curcumin [32]. Another curcumin complex with rare earth metal (rare earth(III) nitrate) also exhibited strong antibacterial activity compared to that of curcumin alone [50]. A complex of silver nanoparticles with curcumin has not only shown high aqueous solubility but also increased antibacterial activity. Thus, this complex presented a capability of preventing infections of wound surfaces caused by bacteria [20]. The Cu^2+^–curcumin complex also exhibits a favorable microbicidal activity and thus has been suggested to be developed into a vaginal microbicidal gel against viral infections [33]. Another study showed that Cu^2+^–curcumin complexes showed higher growth inhibition of *P. aeruginosa* compared to free curcumin, as the minimum inhibitory concentration (MIC) of Cu^2+^–curcumin complexes and free curcumin was 62.5 and 125 µg/mL, respectively. Moreover, Cu^2+^–curcumin complexes (62.5 µg/mL) reduced bacterial cell growth [34].

Along with microbial inhibitory effects, metal–curcumin complexes inhibit bacterial biofilm formations. In one study, an orthovanadium–curcumin complex was evaluated for its potential inhibitory effect on bacterial biofilm formation. This complex inhibited Gram (+) bacteria *S. aureus* and Gram (−) *E. coli* culture-associated biofilms, which was found to be positively correlated with the inhibition of bacterial alkaline phosphatase activity as shown by in silico studies [35]. Another study showed that a Cu^2+^–curcumin complex at a concentration of 1/4 MIC on *P. aeruginosa* highly inhibited biofilm formation, swarming and twitching motilities, and alginate and pyocyanin production, thus indicating its efficacy for the therapy and management of *P. aeruginosa* infections [34]. Curcumin–ruthenium complex exhibited strong antibiofilm activity compared to some FDA approved drugs [31]. In addition to these, the Zn^2+^–curcumin complex has also exhibited potency against biofilm formation [30]. These studies indicate that curcumin–metal complexes exert antimicrobial activity through inducing toxicity, suppressing growth, and inhibiting biofilm formation.

### 4.4. Antiviral

Curcumin exerts antiviral activity through multiple mechanisms. In HIV, it inhibits HIV protease, integrase, Tat transactivation, inflammatory molecules, and various HIV-associated kinases [45]. Metal complexed curcumin has shown better efficacy against viruses compared to free curcumin. Complexes of the central dihydroxy groups of curcumin with boron have exerted higher efficacy over free curcumin as have been shown to decrease the IC50 value to 6 μM as compared to values of 100 μM (HIV-1) and 250 μM (HIV-2) with free curcumin. The boron complexes of curcumin also inactivate HIV proteases [36]. Also, the antiviral activity of a Cu^2+^–curcumin complex was demonstrated against multiple viruses, including herpes simplex virus strains, vesicular stomatitis virus, vaccine virus, coxsackie virus b4, para-influenza-3 virus, respiratory syncytial virus, reovirus-1, sindbis virus, and punta toro virus. The antiviral EC50 value was found to be 4 μg/mL against all the viral strains except for coxsackie virus B4, vesicular stomatitis virus, or respiratory syncytial virus, where it was found to be 0.08 μg/mL for these particular cell cultures. Thus, the Cu^2+^–curcumin complex possesses desirable antiviral activity against various viruses [33].

## 5. Prevention and Treatment of Chronic Inflammatory Diseases by Metal–Curcumin Complex

**Figure 5:**
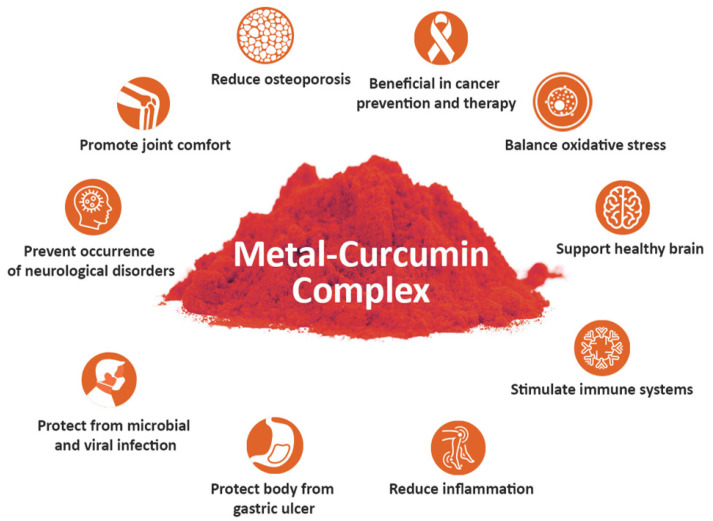
Beneficial health effects and preventive and therapeutic effects of curcumin–metal complexes against various diseases and disorders.

Metal–curcumin complexes demonstrated better antioxidant, anti-inflammatory, antimicrobial, and antiviral effects in preclinical and clinical studies compared to curcumin alone. The properties of metal–curcumin complexes have been manifested into multiple beneficial health effects (Figure 5). The preventive and therapeutic effects of metal–curcumin complexes against chronic inflammatory diseases are summarized in Table 2.

### 5.1. Cancer

Curcumin is reported to be a potent anticancer compound. It acts as a preventive as well as a therapeutic agent against multiple cancer types. Because of curcumin’s physical limitations, metal–curcumin complexes have been investigated in cancer applications. It has been found that curcumin–metal complexes with liposomes present enhanced cellular uptake and ROS generation in cancer cells and thus cause increased cytotoxicity. Cu^2+^–curcumin complexes with liposomes have been shown to increase the therapeutic effects for primary and metastatic breast cancer by improving the stability of curcumin, promoting apoptosis, and inhibiting proliferation and angiogenesis [51]. The Cu^2+^–curcumin complex also induced DNA photocleavage, photocytotoxicity, and cellular localization in HeLa and MCF-7 cancer cells. This complex has shown high photocytotoxicity with low toxicity in the dark and thus exhibits remarkable photodynamic effects [52]. The antitumor effects of a synthetic curcumin–Cu^2+^ complex has also been examined. An enhanced antitumor activity was found with copper chelates of synthetic curcuminoids. It also exhibited activity in enhancing the life span of animals bearing ascites tumors and showed a decrease in solid tumor volume in mice [53]. The encapsulation of the Cu^2+^–curcumin complex in liposomes was also found to improve its antitumor property without any adverse side effects in an animal model of triple negative breast cancer [54]. Thus, Cu^2+^ complexes of curcumin enhance drug delivery and increase the therapeutic efficacy of curcumin.

Another complex with zinc in the form of ZnO-3-mercaptopropionic acid–curcumin was shown to increase the solubility and delivery of curcumin. In addition, this complex exhibited more cytotoxicity towards breast cancer cells compared to free curcumin [55]. Zn^2+^–curcumin complexes were also found to induce cytotoxicity in prostate cancer and neuroblastoma cell lines [56]. Zn^2+^–curcumin complexes have been reported to synergistically promote mitochondrion-mediated apoptosis [57]. Zn^2+^–curcumin complexes not only reduce the viability of cancer cells but also enhance cell death responses to therapeutic drugs such as doxorubicin in vitro and in vivo. It has been shown that this complex degrades and releases curcumin and Zn^2+^ ions inside the cells after internalization into the cells [58]. The Zn^2+^–curcumin complex also induced conformational changes in mutated p53 (R175H and -R273H) proteins and restored the apoptotic function in cancer cells. This complex crossed the blood–tumor barrier and reached the glioblastoma tissues of an orthotopic murine model and caused regression of tumor growth [86].

Ru^2+^–curcumin complexes have also shown a cytotoxic effect in various types of cancer cells at low concentrations [87]. A complex of Ru^2+^–polypyridyl with curcumin has demonstrated higher antiproliferative activity and cytotoxicity against various cancer cells when compared to either free curcumin or cisplatin individually. This complex causes apoptosis in cancer cells through the DNA interaction as well as by inhibiting MEK/ERK signaling [59]. Another ruthenium(II)–letrozole complex with curcumin caused cancer cell death, likely through autophagy [60]. It has also been shown that the Ru^2+^–curcumin complex induces apoptosis more than free curcumin through the inhibition of proteasomes in colon cancer cells. The Ru^2+^–curcumin complex also suppresses isolated proteasomal activities more effectively than free curcumin [61]. To further increase the anticancer activity of Ru^2+^–curcumin complexes, a derivative of curcumin (replaced OH groups with OCH_3_ in curcumin) was used. The resulting curcumin derivative Ru^2+^ complex enhanced antitumor activity over free curcumin [88].

The complex of Mn^2+^–curcumin has revealed potent cytotoxicity in various cancer cell lines, such as HCT-15, SKLU-1, and MCF-7. The IC50 value of the Mn^2+^–curcumin complex was found to be lesser than those of cisplatin and much less than those of free diacetylcurcumin [44]. Thus, considering the antiproliferative and cytotoxicity potential of this complex in human cancer cell lines, a therapeutic potential can be envisioned. The Co^3+^–curcumin complex has also been shown to be biologically more effective against cancer than free curcumin. It was found that the association of Co^3+^ with curcumin enhances the hydrolytic stability of curcumin and results in an enhanced cellular uptake and photo-induced cytotoxicity. It also displays a remarkable photodynamic therapeutic effect in visible light in MCF-7 cells but is much less toxic in the dark. It has been observed that the released curcumin acts as a phototoxin, producing intracellular ROS that causes apoptosis in cancer cells [62]. Because of the high cellular uptake of metal–curcumin complexes, Co^3+^–curcumin complexes have been utilized for their cellular delivery in hypoxic tumor cells, where they are released by the reduction of metal and act as a cytotoxin [56].

The cytotoxic effects of the curcumin and palladium(II) complex have been investigated in A549 and H1299 non-small-cell lung cancer cell lines. The Pd^2+^–curcumin complex has been shown to enhance cytotoxic activity and apoptosis compared to the individual agent [63]. The Pd^2+^–curcumin complex also inhibited cell growth and induced apoptosis in human prostate cancer cells. This complex causes apoptosis through the production of ROS, mitochondrial membrane depolarization, induction of Bax, reduction of Bcl-2 proteins, and JNK phosphorylation in prostate cancer cells [64]. The Pd^2+^ complex with curcumin derivative also exhibited a strong in vitro antitumor effect against human colorectal carcinoma and inhibited its hepatic metastasis. Furthermore, this complex has been shown to decrease tumor cell membrane expression of prominin-1 (CD133) molecules and restrict stem cell factor (SCF) release [65], which indicates its mechanism of antitumorigenesis and antimetastasis.

Ni^2+^–curcumin complexes display anticancer effects in multiple cancer cells, including human cervical carcinoma and lung cancer cells, mainly by cell cycle arrest, production of ROS, and loss of mitochondrial membrane potential [66]. Gallium–curcumin and gallium–diacetylcurcumin complexes have also exhibited cytotoxic effects on bladder, breast, and prostate carcinoma cell lines, indicating their potential for cancer treatment [24]. The complex of vanadium with curcumin also improves the therapeutic efficacy of curcumin. Additionally, this complex exhibited profound photodynamic therapy effect in HeLa and MCF-7 cancer cells in visible light, with less toxicity in the dark [72]. Other than HeLa cells, oxovanadium(IV)–curcumin complexes exhibit photocytotoxicity in hepatic Hep G2 cancer cells in visible light. This complex increases the cellular uptake of curcumin in cancer cells, further causes the formation of ROS due to light, and results in cancer cell apoptosis [73].

Platinum–curcumin is another complex which has shown phototoxic and apoptosis-inducing effects in cancer cells. This complex was found to induce cellular ROS and further apoptotic cell death under visible light [67,68]. Furthermore, this complex was found to synergistically enhance the chemotherapeutic effect of drugs and to sensitize cisplatin-resistant A549/DDP cells [69]. Upon exposure to visible light, it also forms a platinum-bound DNA adduct and leads to photocytotoxicity in cancer cells [70]. The Pt^2+^–curcumin complex also overcomes the side effects of other platinum-based chemotherapy. In one study, the platinum–curcumin complex nanoparticles displayed enhanced anticancer effects for both in vitro and animal models with reduced side effects. In addition, it inhibited the PI3K/AKT signaling pathway and suppressed the expression of vascular endothelial growth factor-2 (VEGFR2) and matrix metalloproteinase-2 (MMP2) molecules, which resulted in its increased antimetastatic activity [71].

Iron oxide nanoparticle formulations of curcumin also exhibit anticancer effects and have been found to have nontoxic, bioactive, and anti-inflammatory effects and to enhance drug delivery to tumors. If treated with gemcitabine, these formulations increase gemcitabine uptake due to their ability to increase human nucleoside transporter genes (DCK, hCNT) and decrease ribonucleotide reductase subunits (RRM1/RRM2). Iron oxide nanoparticle–curcumin complexes affect the tumor microenvironment through the inhibition of Sonic Hedgehog (SHH) signaling and blocking the oncogenic CXCR4/CXCL12 signaling pathway. In a mouse model, this complex was shown to enhance accumulation of curcumin in the pancreas, which further potentiated gemcitabine-induced tumor growth and metastasis reduction [74]. Moreover, an iron–curcumin complex was shown to induce apoptosis and inhibit invasion of breast cancer cells [75]. As metal generates electromagnetic fields, a curcumin loaded nanoparticle with Fe_3_O_4_ was prepared and tested against leukemia HL-60 cells. It was found that this formulation increased contrast magnetic resonance, resulting in high apoptosis rates [76]. Thus, iron complexation may be considered as a strategy for improving the potency of curcumin in the therapy of cancer.

### 5.2. Arthritis

Curcumin has been found to have high efficacy as a preventive and therapeutic agent for various types of diseases and disorders. Subsequently, metal complexes of curcumin were also challenged against various human diseases to determine their comparative efficacy with curcumin. In a study, a vanadyl–curcumin complex was used against arthritis. It was found that the vanadyl–curcumin complex suppressed the proliferation of synoviocyte and growth of smooth muscle cells and mouse lymphoma cells more effectively than curcumin alone. As it inhibits synoviocyte proliferation without causing any toxicity, it can be used for the therapy of rheumatoid arthritis [77]. Another complex of curcumin with gold has been analyzed in an animal model of arthritis. Treatment with gold(I)–curcumin complex (30 mg/kg/day by injection) caused remission in adjuvant-induced polyarthritis as indicated by a reduction in paw swelling after 3 weeks of administration. This complex also improved the anatomical changes occurred in the affected limbs of rats [78].

### 5.3. Osteoporosis

Metal complexes of curcumin have also been found to be effective in osteoporosis. As curcumin prevents osteoporosis by inhibiting osteoclastogenesis (bone resorption) and suppressing osteoclast-inducing mediators [89], the antiosteoporotic effects of metal complexes of curcumin have been investigated. Some metals, such as lanthanide ions, have shown a high affinity for bone and inhibit the formation of osteoclasts. As a result, a complex of curcumin with lanthanide Ln(Curc)3 was created as a potential treatment for osteoporosis. The antiosteoporotic activity of this complex was investigated in an osteoblast-like MG-63 cell line derived from a human osteosarcoma. A promising toxicity toward MG-63 cells was found by lanthanide curcumin complex treatment [79]. A complex of gold nanoparticles and curcumin was also examined for its antiosteoporotic activity. It was found that this complex inhibits the receptor activator of NF-κB ligand (RANKL)-induced osteoclastogenesis in bone marrow-derived macrophages. Curcumin–gold nanoparticles also inhibited osteoclast differentiation markers such as c-Fos, TRAP, nuclear factor of activated T cells 1 (NFATc1), and osteoclast-associated receptor (OSCAR). In addition to in vitro models, the curcumin–gold nanoparticle complex also prevented bone loss and improved bone density in an ovariectomy-induced osteoporosis mouse model [80]. Thus, curcumin metal complexes could be useful agents for the prevention and treatment of osteoporosis.

### 5.4. Neurological Disorders

The nervous system of the brain controls thoughts, memory, movement, and emotions by a series of complex functions. However, genetic disorders, congenital abnormalities, infections, lifestyle, environmental factors, and brain injury may lead to neurological disorders such as Alzheimer’s disease, Parkinson’s disease, dementia, schizophrenia, and depression. Some neurological disorders are connected to the aggregation of tau protein and deposition of β-amyloid plaques in the neurons. Among various natural compounds, curcumin has demonstrated beneficial effects on brain health through several mechanisms. As curcumin is a strong antioxidant and anti-inflammatory agent, it can maintain brain health and can prevent the occurrence of neurological disorders. Curcumin also binds to amyloid β-protein, inhibits tau protein, causes metal chelation, increases neurogenesis activity, and promotes synaptogenesis [90]. However, its poor bioavailability limits the therapeutic efficacy of curcumin.

Accumulated evidence indicates that metal ions such as Al^3+^, Mn^2+^, Fe^3+^, Cu^2+^, Pb^2+^, Hg^2+^, As^3+^, and Zn^2+^ are potential risk factors in developing neurodegenerative diseases [91]. The Al^3+^ ion has been shown to be the most harmful for the brain, as it is involved in neural fibrillation and β-amyloid plaque formation [92]. Since curcumin has the ability to cross the blood–brain barrier due to its hydrophobic nature, it can strongly chelate the metal ions in the brain and prevent metal-induced neurotoxicity. Due to the increased stability and bioavailability of curcumin in metal–curcumin complexes, the use of metal–curcumin complexes against metal-induced neurotoxicity has been investigated by several studies. It has been shown that gadolinium–curcumin inhibits amyloid-β plaque aggregation more than the free metal or Zn^2+^-induced analogues [81]. Furthermore, Liu [82] showed that the Ru^2+^ –curcumin complex has a stronger ability to inhibit the aggregation of tau peptide, thus providing a strategy to design anti-Alzheimer’s drugs with curcumin.

In an in vitro study, Yan et al. [21] noted the neuroprotective effects of the complexes of curcumin with Cu^2+^ or Zn^2+^ on hydrogen peroxide (H_2_O_2_)-induced injury in rat PC12 cells. Curcumin–Cu^2+^ or –Zn^2+^ complex systems combat oxidative stress by enhancing catalase, superoxide dismutase, and glutathione peroxidase activities and attenuating increased level of MDA. These complexes also inhibited neuronal cell death by downregulating the NF-κB signaling pathway and upregulating Bcl-2/Bax molecules. Cu^2+^–curcumin systems have been shown to be more protective than the curcumin–Zn^2+^ systems and much more effective than unchelated curcumin. Thus, curcumin–Cu^2+^ or –Zn^2+^ complex systems possess significant neuroprotective effects [21]. Further in vivo studies conducted on Swiss albino mice showed that Fe^3+^–curcumin complexes have the ability to reduce the accumulation of β-amyloid_25–35_ protein. In addition, the Fe^3+^–curcumin complex displayed a strengthening of memory in mice. The Fe^3+^–curcumin complex was found to be more effective than curcumin or the Mn^2+^–curcumin complex [83]. These studies indicate that metal complexes of curcumin could offer new avenues in the maintenance of brain health as well as in the prevention and therapy of neurological disorders.

### 5.5. Other

Curcumin–metal complexes also have better protective efficacy against gastric ulcers than free curcumin. In one study, oral treatment with a Zn^2+^–curcumin complex blocked the ethanol-induced formation of ulcer lesions and thus exhibited gastroprotective activity in rats. In addition, curcumin–Zn^2+^ complexes enhanced the growth of gastric fibroblast cells more prominently than free curcumin at the same doses [25] and thus aided in recovery from gastric ulcers. Other studies also confirmed that oral treatment with a curcumin–Zn^2+^ complex in a rat model reduced gastric volume, gastric lesions, free acidity, total acidity, and pepsin more effectively than curcumin alone [26]. As curcumin has demonstrated antidiabetic effects when administered alone, the Zn^2+^–curcumin complex showed better efficacy than free curcumin. In a study, oral treatment with a Zn^2+^–curcumin complex exerted a hypoglycemic effect in a streptozotocin-induced diabetic rat model better than free curcumin. A decrease in blood glucose, glycosylated hemoglobin (Hb)A1c, and lipid profile parameters and an improvement in plasma insulin levels have been observed in rats receiving Zn^2+^–curcumin treatment. This complex also displayed nontoxicity as it reduced activities of serum aspartate aminotransferase (AST), alanine aminotransferase (ALT), creatinine, and urea in diabetic rats [84]. Furthermore, Zn^2+^–curcumin complexes have shown their ability to protect against reproductive system impairments. In one study, oral treatment with a Zn^2+^–curcumin complex suppressed cyclophosphamide-induced increase of oxidative stress in mouse testis. A curcumin–Zn^2+^ complex has also been shown to restore a cyclophosphamide-induced decrease in body and reproductive organ weights. This complex further ameliorated reproductive system impairments by improving sperm parameters (sperm count, viability, motility) and decreasing serum testosterone. Curcumin–Zn^2+^ has shown better efficacy in improving cyclophosphamide-induced reproductive injury compared to curcumin at the same dose. These results suggest that curcumin– Zn^2+^ has a better efficacy in protecting reproductive damage than curcumin alone [85].

## 6. Application of Metal–Curcumin Complex on Biological Imaging and Radioimaging

As curcumin is a fluorescent molecule, metal-curcumin complexes may be used in biological imaging and radioimaging. It has been reported that materials with large two-photon absorption cross sections are better for the bioimaging of living cells and tissues [93]. As curcumin complexes with copper have properties of higher quantum yield and larger two-photon absorption, they have been investigated for biological imaging and radioimaging. In one study, cells were imaged in vitro and in vivo by two-photon fluorescence microscopy, and it was found that the Cu^2+^–curcumin complexes have a high tumor targeting capability and good photostability. Thus, it is suggested that these complexes could be promising probes for in vivo imaging and can be potentially useful for early tumor detection [94].

Ni^2+^–curcumin complexes have shown an intense curcumin-based band at ∼440 nm in DMSO-Tris-HCl buffer. These complexes interact with human serum albumin with moderate affinity and demonstrate substantial in vitro light-induced cytotoxicity in cancer cells [66]. Palladium complexes of curcumin have also been shown to be helpful in fluorescence imaging of cells that are useful in studying the delivery of curcumin into cancer cells and retention of its potential anticancer activity [95]. The complex of curcumin and gadolinium ion (Gd^3+^) metal self-assembled with sodium dodecyl sulfate and HEPES caused an increase in fluorescence quantum. Thus, the increased cellular uptake of curcumin resulted in an enhanced fluorescence image [96]. Other complexes, such as boron–curcumin and iron–curcumin complexes, have shown high fluorescence efficiency (quantum yield) and greater photostability in solution. Thus, the higher photostability and larger quantum yields of these complexes may make them good candidates for medical imaging and in vitro studies [97].

A theranostic modality was also carried out by using a boron–curcumin complex (RbCur). For this, the RbCur complex and gadolinium metal were delivered simultaneously into tumor cells followed by a boron and gadolinium neutron capture therapy (NCT). Furthermore, magnetic resonance imaging (MRI) was performed to determine the internalization of boron and gadolinium by tumor cells. This complex not only helped in the diagnosis of drug internalization but also in the determination of the cytotoxic activity of curcumin in cancer cells [98]. Another theranostic study was done by using magnetic Fe_3_O_4_ nanoparticles encapsulated in a silica shell loaded with curcumin. Both imaging and therapeutic functions of the complex were performed by a set of microscopy, spectroscopy, and biochemical methods in leukemia HL-60 cells. It displayed high apoptosis rates and contrast magnetic resonance images, indicating its success in theranostic regimens [76]. Other complexes with lanthanide(III) have shown remarkable photocytotoxicity in HeLa cells in visible light and have also become helpful in the localization of drugs in HeLa cells by confocal imaging [99]. Oxovanadium(IV) complexes of curcumin have also exhibited photocytotoxicity in visible light. As curcumin–oxovanadium complexes emit green fluorescence, they have been used for cellular imaging [87]. Furthermore, curcumin–oxovanadium complexes have aided in determining cellular uptake of the complexes as revealed by fluorescence microscopic studies [73].

## 7. Solubility, Stability, and Cellular Uptake of Metal–Curcumin Complexes

Curcumin possesses a wide range of biological activities. However, its hydrophobic nature, low-intestinal absorption, rapid metabolism, and systemic elimination cause a reduction in curcumin’s bioavailability and subsequently limit its clinical use [100]. So far, many approaches have been implemented to improve the water solubility and bioavailability of curcumin. In past decades, complexes of curcumin with metals have been synthesized. As curcumin bears 1,3-diketones with keto-enol isomerization in its chemical structure, it can readily form a complex with various metal ions, such as Mn^2+^, Fe^2+^, Cu^2+^, Zn^2+^, Al^3+^, and Fe^3+^ [101], which may help with the solubility and stability of curcumin.

Studies demonstrate that curcumin–metal complexes have enhanced solubility, stability, bioavailability, and biochemical activities compared to curcumin alone [101,102]. In one study, the stability of curcumin with its complexation with divalent metal ions, such as Zn^2+^, Cu^2+^, Mg^2+^, and Se^2+^, was investigated. The in vitro stability results showed that all complexes have a higher stability compared to free curcumin [103]. In another study, the in vitro kinetic degradation, stability, and solubility of the Zn^2+^–curcumin complex was analyzed spectrophotometrically. It was found that the Zn^2+^–curcumin complex exhibited good stability and higher solubility with better pharmacodynamic effects than free curcumin [104]. It was also observed that Zn^2+^–curcumin displays a higher stability than curcumin in buffered media, but its degradation begins with the increase of pH [105]. A complex of copper and curcumin was synthesized, and to further increase its aqueous solubility, β-cyclodextrin (CD) was included in the complex. This Cu^2+^–curcumin–CD complex resulted in a high aqueous solubility of the curcumin [106]. Curcumin interacts through the OH group of CD and the phenolic hydroxyl group of the curcumin. Thus, in this curcumin–metal complex coated by CD, the solubility of curcumin in water increases [107].

Liposome-loaded metal ions and curcumin complexes have demonstrated an increased stability of curcumin. A release profile of curcumin–Zn^2+^/Cu^2+^ liposomes was conducted in PBS with or without EDTA and showed that curcumin– Zn^2+^/Cu^2+^ liposomes have sustained release profiles. Cu^2+^–curcumin complexes were more stable than Zn^2+^–curcumin complexes because the stability constant (K) of Cu^2+^–curcumin complexes is significantly higher than that of Zn^2+^–curcumin [51]. However, the interaction energies reveal that Zn^2+^–curcumin complexes are more stable than the Mn^2+^–curcumin and Fe^2+^–curcumin complexes [7]. The curcumin was shown to dissociate from Zn^2+^–curcumin complexes, while the Cu^2+^–curcumin complexes slowly release from the curcumin–Cu^2+^ complex liposomes [51].

Curcumin–barium (Ba^2+^) complexes have been shown to improve the stability of curcumin by greater than 50% as detected by UV-vis spectroscopy. The stability of curcumin was found to be further increased to approximately 70% in water by the loading of the Ba^2+^–curcumin complex nanoparticles into pluronic micelles. It has found that Ba^2+^ interacts predominantly through di-phenolic groups of curcumin to form an end-to-end complex. These Ba^2+^–curcumin supramolecule nanoparticles showed improved solubility and stability [108]. Another complex of curcumin with palladium metal was synthesized as a tetrafacial water-soluble molecular barrel to increase the solubility and stability of curcumin. Curcumin in this complex was found to be highly stable, as the barrel encapsulates curcumin inside a molecular cavity and protects it from photodegradation. This complex also increased solubility and cellular uptake as compared to free curcumin in water [95].

Curcumin with silver nanoparticle formates also increases the solubility and stability of curcumin in complexes. Curcumin reduces and caps the silver nanoparticles, which increases its stability and solubility in water [109]. Oxidovanadium(IV) complexes of curcumin have also shown aqueous solubility and stability of curcumin in a solution phase over a long period of time of 48 h when chloride salt metals were used in the synthesis of the complex [72]. Banerjee et al. [87] further reported that the binding of curcumin to the oxovanadium moiety resulted in stability against any hydrolytic degradation of curcumin. A Mn^2+^–curcumin complex was also synthesized to determine the hydrolytic stability of the complex. It was found that the Mn^2+^–curcumin complex is stable in physiological buffers and media, as opposed to curcumin, even under reducing conditions [32]. The gallium complexed curcumin increases the stability, cellular uptake, and bioavailability of curcumin in both in vitro and in vivo cancer models [110]. In a study, a gallium–curcumin complex and gallium-diacetylcurcumin showed a higher uptake by colorectal carcinoma (HT29) and lymphoma (K562) cell lines than in lymphocytes [111].

The incorporation of curcumin into the self-assembled complex of gadolinium metal ion (Gd^3+^), sodium dodecyl sulfate, and HEPES causes an increase in the stability of curcumin. The incorporation of curcumin and curcumin borondifluoride into the self-assembled complex showed an approximately 50% and 30% improved stability, respectively [96]. Boron–curcumin and iron–curcumin complexes have shown superior photostability in solution. This increased photostability was found to be attributed to the coordination structures and the removal of β-diketone group in curcumin [97]. Cobalt also increases stability of curcumin by binding to its enolic form. Binding to cobalt(III) increases the hydrolytic stability of curcumin and significant cellular uptake and bioactivity compared to free curcumin [62]. Along with cellular uptake, this metal complex profoundly influences the distribution and release mechanism of curcumin [112]. Moreover, platinum–curcumin complexes showed stability over a study period of 48 h [68]. Additionally, platinum-based nanoparticle–curcumin complexes have been shown to greatly improve the solubility as well as stability of curcumin [69]. Further study confirmed that the platinum–curcumin complex reduces the hydrolytic instability of curcumin [70].

## 8. Conclusions

The ability of certain metals to complex with curcumin is clearly a better way for treatment modalities for any number of health conditions found in humans and animals than the traditional approaches with curcumin alone. Curcumin–metal complexes have been shown to have superior antioxidant, anti-inflammatory, antimicrobial, and antiviral activities than curcumin alone. These curcumin–metal complexes can be used to treat cancer, arthritis, osteoporosis, neurological disorders, and a host of other diseases and health conditions that afflict humans and animals. Certain metals will work more effectively for certain health conditions than others. Determining which metals combined with curcumin have more beneficial effects for certain diseases is the subject of future research. This review has detailed many particular curcumin–metal complexes for specific conditions, such as the use of copper– and zinc–curcumin complexes for both antioxidant activity and anti-inflammatory activity; the use of copper–curcumin complexes for antimicrobial activity; the use of boron–curcumin complexes for antiviral activity; the use of copper–, zinc–, and ruthenium–curcumin complexes for cancer; the use of lanthanide–curcumin complexes for osteoporosis; or the use of copper–, zinc–, or iron–curcumin complexes for neurological disorders.

The question of which metal complex will be the most effective for any particular health use remains to be answered. However, zinc–curcumin complexes, in particular, may prove to be useful in many health conditions related to oxidation and inflammation. Oxidation plays an underlying role in many inflammatory conditions. Both zinc and curcumin are known to play beneficial roles in the immune system in relation to oxidation and inflammation. Zinc is a key requirement in maintaining immunity for enzymes needed in immune functions. For example, it is known that zinc content is significantly lower in rheumatoid arthritis (RA) patients as compared to healthy individuals [113]. The lowest levels are associated with more severe disease. Zinc may help to improve RA symptoms by supporting the immune system and cartilage. Zinc also has the potential to be a normalization supplement for osteoarthritis in humans [114] by modulating cartilage degradation. As such, the use of zinc–curcumin complexes may prove useful for inflammatory conditions, allowing for the provision of the required zinc along with the benefits of curcumin. In addition, as zinc possesses antiviral activity [115], the use of zinc–curcumin complexes may also prove especially useful for viral diseases.

While the various specific metals used in these curcumin complexes continue to be researched for optimal efficacy, the future of these curcumin–metal complexes undoubtedly shows great promise for better treatment and prevention in a wide variety of health conditions.

## Figures and Tables

**Table 1:** Pharmacological effects of metal–curcumin complexes.

Metal–Curcumin Complex	Effects	References
**Antioxidant**		
Zinc–curcumin	Increases antioxidant enzymes and decreases MDA level caused by cold-restraint stress in mice	[17]
Iron–curcumin	Improves antioxidant activity	[18]
Gold NP–curcumin	Shows good antioxidant activity	[19]
Silver–curcumin	Presents good antioxidant activity and cell compatibility	[20]
Copper–curcumin Zinc–curcumin	Enhances the superoxide dismutase, catalase, and glutathione peroxidase activities and attenuates MDA levels in PC12 cells	[21]
Copper–curcumin	Exhibits antioxidant potential by scavenging DPPH radicals	[11]
Zinc NP–curcumin	Exerts antioxidant activity as it scavenges ABTS+ and DPPH free radicals	[22]
Ruthenium–curcumin	Exhibits potent antioxidant activity	[23]
Gallium–diacetylcurcumin	Presents an increase in the antioxidant efficiency	[24]
**Anti-inflammatory**		
Zinc–curcumin	Suppresses NF-κB, TGF-β1, IL-8, IL-6, TNF-α in rat model	[25,26]
Copper–curcumin	Suppresses PKCδ and NF-κB	[27]
Zinc–curcumin Copper–curcumin	Inhibits H_2_O_2_-induced NF-κB in PC12 cells	[21]
Gadolinium–curcumin	Inhibits pIKKα, pIKKα/β, and NF-κB in pancreatic cancer cells	[28]
**Anti-microbial**		
Cobalt–, Nickel–, Copper–curcumin	Inhibits growth of *E. coli*, *S. aureus*, *K. pneumoniae*, *S. pyogenes*, and *P. aeruginosa*	[29]
Zinc–curcumin	Inhibits growth of *P. aeruginosa*	[30]
Zinc–curcumin	Inhibits growth of *B. subtilis*, *E. coli*	[22]
Ruthenium–curcumin	Inhibits growth of drug resistant *S. aureus*, reduces mean bacterial counts in murine model of Staphylococcus infection	[31,32]
Manganese–curcumin	Inhibits growth of *S. aureus*, *E. coli*	[32]
Silver–curcumin	Combats multiple bacteria-induced infections on wound surfaces	[20]
Copper–curcumin	Exhibits antimicrobicidal activity	[33]
Copper–curcumin	Inhibits growth of *P. aeruginosa*	[34]
Orthovanadium–curcumin	Inhibits bacterial biofilm formation, growth of *S. aureus* and *E. coli*	[35]
Copper–curcumin	Inhibits growth of *P. aeruginosa* and biofilm formation	[34]
Ruthenium–curcumin	Exhibits strong antibiofilm activity	[31]
Zinc–curcumin	Exerts potency against biofilm formation	[30]
**Antiviral**		
Boron–curcumin	Inactivates HIV proteases	[36]
Copper–curcumin	Inhibits multiplication of multiple types of viruses	[33]

MDA—Malondialdehyde; NP—Nanoparticle; NF-κB—Nuclear factor-kappaB, IL—Interleukin; TGF-β1—Tumor growth factor beta1; PKCδ—Protein kinase C delta; pIKK—Phosphorylated inhibitory kappaB kinase.

**Table 2:** Prevention and treatment of chronic inflammatory diseases by metal–curcumin complexes.

Metal–Curcumin Complex	Models	Mechanisms	References
**Cancer**			
Copper–curcumin	Breast cancer cells	Promotes apoptosis, inhibits proliferation and angiogenesis	[51]
Copper–curcumin	Cervical and breast cancer cells	Induces photocytotoxicity	[52]
Copper–curcumin	Mice	Increases life span of ascites tumor bearing animals and reduces tumor growth	[53]
Copper–curcumin	Murine	Inhibits growth of human breast tumors in animals	[54]
Zinc–curcumin	Breast cancer cells	Induces more cytotoxicity compared to free curcumin	[55]
Zinc–curcumin	Prostate cancer, neuroblastoma cell lines	Induces cytotoxicity	[56]
Zinc–curcumin	In vitro and in vivo	Promotes mitochondria-mediated apoptosis	[57]
Zinc–curcumin	In vitro and in vivo	Enhances cell death response to therapeutic drugs like doxorubicin	[58]
Ruthenium–curcumin	Various cancer cells	Induces cytotoxic effect	[56]
Ruthenium–curcumin	Various cancer cell lines	Displays antiproliferative activity and causes apoptosis via DNA interaction and inhibiting MEK/ERK signaling pathway	[59,60]
Ruthenium–curcumin	Glioblastoma and breast cancer cells	Induces cell death likely through autophagy	[60]
Ruthenium–curcumin	Colon cancer cells	Induces apoptosis through the inhibition of proteasomes	[61]
Cobalt–curcumin	Breast cancer cells	Acts as a phototoxin, generates intracellular ROS and causes apoptosis	[62]
Palladium–curcumin	Lung cancer cells	Induces cytotoxicity and apoptosis	[63]
Palladium–curcumin	Prostate cancer cells	Inhibits cell growth and induces apoptosis via ROS, mitochondrial pathway, and JNK phosphorylation	[64]
Palladium–curcumin	Colorectal carcinoma	Exhibits antitumor activity through decrease of prominin-1 (CD133) and SCF release	[65]
Manganese–curcumin	Colon, breast, and lung cancer cells	Causes cytotoxic effects	[44]
Nickel–curcumin	Cervical carcinoma, lung cancer cells	Exerts anticancer activity by cell cycle arrest, production of ROS, and mitochondrial membrane depolarization	[66]
Gallium–curcumin	Bladder, breast, and prostate carcinoma cells	Exhibits cytotoxic effect	[24]
Platinum–curcumin	Cervical, liver, breast, and lung adenocarcinoma	Causes phototoxic and apoptosis through cellular ROS	[67,68]
Platinum based NP–curcumin	Drug resistant A549/DDP cells	Synergistically enhances the chemotherapeutic effect of drugs	[69]
Platinum–curcumin	Cancer cells	Induces apoptotic photocytotoxicity through platinum-bound DNA adducts	[70]
Platinum–curcumin	Non-small-cell lung cancer cells	Exhibits anticancer & antimetastatic activity by inhibiting PI3K/AKT, MMP2 and VEGFR2	[71,72]
Oxidovandium–curcumin	Cervical and breast cancer cells	Shows photodynamic therapy effect	[72]
Oxovanadium–curcumin	Liver cancer cells	Induces apoptosis through ROS generation	[73]
Iron–curcumin	Pancreatic cancer cells	Targets tumor microenvironment via suppression of SHH pathway and CXCR4/CXCL12 signaling	[74]
Iron–curcumin	Breast cancer cells	Induces apoptosis and inhibits invasion	[75]
Iron–curcumin	Leukemic cells	Increases contrast magnetic resonance resulted in high apoptosis	[76]
**Arthritis**			
Vanadium–curcumin	In vitro	Inhibits smooth muscle cell growth, synoviocyte proliferation, and mouse lymphoma cell growth	[77]
Gold–curcumin	Rats	Causes remission in adjuvant induced polyarthritis and reduces paw swelling	[78,79]
**Osteoporosis**			
Lanthanide–curcumin	MG-63 cells	Induces toxicity toward MG-63 cells	[79]
Gold NP–curcumin	Bone marrow-derived macrophages and mice	Inhibits RANKL-induced osteoclastogenesis through inhibition of c-Fos, NFATc1, TRAP, and OSCAR; improves bone density and prevents bone loss in mice	[80]
**Neurological disorders**			
Gadolinium–curcumin	Aβ aggregate protein	Inhibits amyloid-β plaques aggregation	[81]
Ruthenium–curcumin	Tau peptides	Inhibits aggregation of tau peptide	[82]
Copper–curcumin Zinc–curcumin	PC12 cells	Inhibits H_2_O_2_-induced neuronal cell death via downregulating NF-κB pathway and upregulating Bcl-2/Bax pathway	[21]
Iron–curcumin	Mice	Reduces accumulation of β-amyloid_25–35_ protein and strengthens memory	[83]
**Other diseases**			
Zinc–curcumin	Rats	Prevents ethanol-induced formation of ulcer lesions	[25]
Zinc–curcumin	Rats	Blocks gastric lesions, reduces gastric volume, free acidity, total acidity, and pepsin	[26]
Zinc–curcumin	Rats	Exerts hypoglycemic effect	[84]
Zinc–curcumin	Mice	Protects reproductive system impairments	[85]

MEK—Mitogen-activated protein kinase; ERK—Extracellular-signal-regulated kinase; ROS—Reactive oxygen species; JNK—c-Jun N-terminal kinase; SCF—Stem cell factor; PI3K—Phosphoinositide 3-kinase; MMPs—Matrix metalloproteinases; VEGFR2—Vascular endothelial growth factor receptor 2; CXCR4—C-X-C chemokine receptor type 4; CXCL2—C-X-C motif chemokine ligand 2; RANKL—Receptor activator of nuclear factor kappa-Β ligand; NFATc1—Nuclear factor of activated T-cells, cytoplasmic 1; TRAP—Tartrate-resistant acid phosphatase; OSCAR—Osteoclast associated receptor.
